# NLRP3 inflammasome and pyroptosis in cardiovascular diseases and exercise intervention

**DOI:** 10.3389/fphar.2024.1368835

**Published:** 2024-04-12

**Authors:** Ping Ding, Yuanming Song, Yang Yang, Cheng Zeng

**Affiliations:** ^1^ Center for Drug Research and Development, Guangdong Pharmaceutical University, Guangzhou, China; ^2^ Zhuhai People’s Hospital, Zhuhai Clinical Medical College of Jinan University, Zhuhai, China; ^3^ Key Specialty of Clinical Pharmacy, The First Affiliated Hospital of Guangdong Pharmaceutical University, Guangzhou, China; ^4^ Guangdong Key Laboratory of Pharmaceutical Bioactive Substances, Guangdong Pharmaceutical University, Guangzhou, China

**Keywords:** cardiovascular disease, exercise, pyroptosis, NLRP3 inflammasome, intervation

## Abstract

NOD-like receptor protein 3 (NLRP3) inflammasome is an intracellular sensing protein complex that possesses NACHT, leucine-rich repeat, and pyrin domain, playing a crucial role in innate immunity. Activation of the NLRP3 inflammasome leads to the production of pro-inflammatory cellular contents, such as interleukin (IL)-1β and IL-18, and induction of inflammatory cell death known as pyroptosis, thereby amplifying or sustaining inflammation. While a balanced inflammatory response is beneficial for resolving damage and promoting tissue healing, excessive activation of the NLRP3 inflammasome and pyroptosis can have harmful effects. The involvement of the NLRP3 inflammasome has been observed in various cardiovascular diseases (CVD). Indeed, the NLRP3 inflammasome and its associated pyroptosis are closely linked to key cardiovascular risk factors including hyperlipidemia, diabetes, hypertension, obesity, and hyperhomocysteinemia. Exercise compared with medicine is a highly effective measure for both preventing and treating CVD. Interestingly, emerging evidence suggests that exercise improves CVD and inhibits the activity of NLRP3 inflammasome and pyroptosis. In this review, the activation mechanisms of the NLRP3 inflammasome and its pathogenic role in CVD are critically discussed. Importantly, the purpose is to emphasize the crucial role of exercise in managing CVD by suppressing NLRP3 inflammasome activity and proposes it as the foundation for developing novel treatment strategies.

## 1 Introduction

Cardiovascular diseases (CVD) remain a prevalent global health concern, causing a significant burden of illness and mortality, with approximately one-third of all deaths attributed to this condition (Mensah et al., 2019). The common symptoms of CVD include chest pain, shortness of breath, irregular heartbeat, fatigue, and decreased physical stamina (Tutor et al., 2023; Wong and Sattar, 2023). CVD encompass various disorders that affect the heart and blood vessels, including atherosclerosis (AS), obesity, diabetes, hyperhomocysteinemia (HHcy), myocardial infarction (MI), hypertension, heart failure (HF), and diabetic cardiomyopathy (DCM) (Konishi et al., 2022; Haidar and Horwich, 2023). The conventional risk factors that are widely recognized for CVD, such as hypertension, hypercholesteremia, diabetes mellitus, and cigarette smoking, remain acknowledged as the main factors responsible for the development and advancement of this condition (Alten et al., 2020).

NOD-like receptor protein 3 (NLRP3) inflammasome is a molecular platform that triggers caspase-1 and facilitates the secretion of interleukin (IL)-1β and IL-18 in response to cellular infection or stress (Valenzuela et al., 2023). This activation results in the cleavage of gasdermin D (GSDMD) by caspase-1, generating an N-terminal GSDMD fragment (Valenzuela et al., 2023). This fragment induces the formation of membrane pores and triggers inflammatory cell death, namely pyroptosis (Valenzuela et al., 2023). The involvement of NLRP3 inflammasome and pyroptosis has been established in cardiovascular risk factors such as hyperlipidemia, diabetes, hypertension, obesity, and HHcy (Alten et al., 2020). Targeting NLRP3 inflammasome activation and pyroptosis holds great potential for therapeutic interventions against CVD.

Extensive research has demonstrated that exercise plays a crucial role in weight management (Murray et al., 2023), blood pressure (BP) reduction (Tucker et al., 2022), blood sugar (Kar et al., 2019) and lipid regulation (Lee et al., 2020), consequently lowering the risk of CVD (Li et al., 2023). Moreover, moderate exercise improves cardiovascular system function and structure, strengthens the heart muscle, enhances cardio-pulmonary function, promotes blood circulation, and increases the heart’s tolerance and overall health (Alten et al., 2020). Furthermore, exercise exhibits a clear anti-inflammatory effect (Lee et al., 2020). It is widely acknowledged that exercise can effectively reduce chronic inflammation by inhibiting the expression of inflammatory factors while increasing the release of anti-inflammatory cytokines (Lee et al., 2020). Previous studies have indicated that exercise can decrease the activation of the NLRP3 inflammasome to substantially inhibit IL-1β, and IL-18 release (Li et al., 2023; Liu et al., 2023). This review mainly focuses on how exercise can improve CVD by influencing NLRP3 inflammasome or pyroptosis. Additionally, this review will investigate exercise as a therapeutic strategy via NLRP3 inflammasome for managing CVD and address the research questions that need to be explored in the future.1.

## 2 Overview of NLRP3 inflammasome

Inflammasomes are crucial components of the immune system that play a major role in initiating inflammatory responses (Chang, 2023). They are composed of sensor proteins known as pattern recognition receptors that oligomerize and form a platform for the activation of caspase-1 in response to damage-associated molecular patterns (DAMPs) or pathogen-associated molecular patterns (PAMPs) (Chang, 2023). The nucleotide-binding domain-like receptor (NLR) family all share a central nucleotide-binding domain, and most members have a C-terminal leucine-rich repeat (LRR) domain and a variable N-terminal domain (Swanson et al., 2019). While certain members such as NLRP1, NLRP3, and NLRC4 are recognized as NLRs capable of forming inflammasomes, others like NLRP6 and NLRP12 are considered potential inflammasome sensors (Swanson et al., 2019). The NLRP3 inflammasome, in particular, is essential for the host’s immune defense against various bacterial, fungal, and viral infections (Dilucca et al., 2021). However, when dysregulated, it has been implicated in the development of several inflammatory disorders, including CVD (Paerewijck and Lamkanfi, 2022).

**FIGURE 1:**
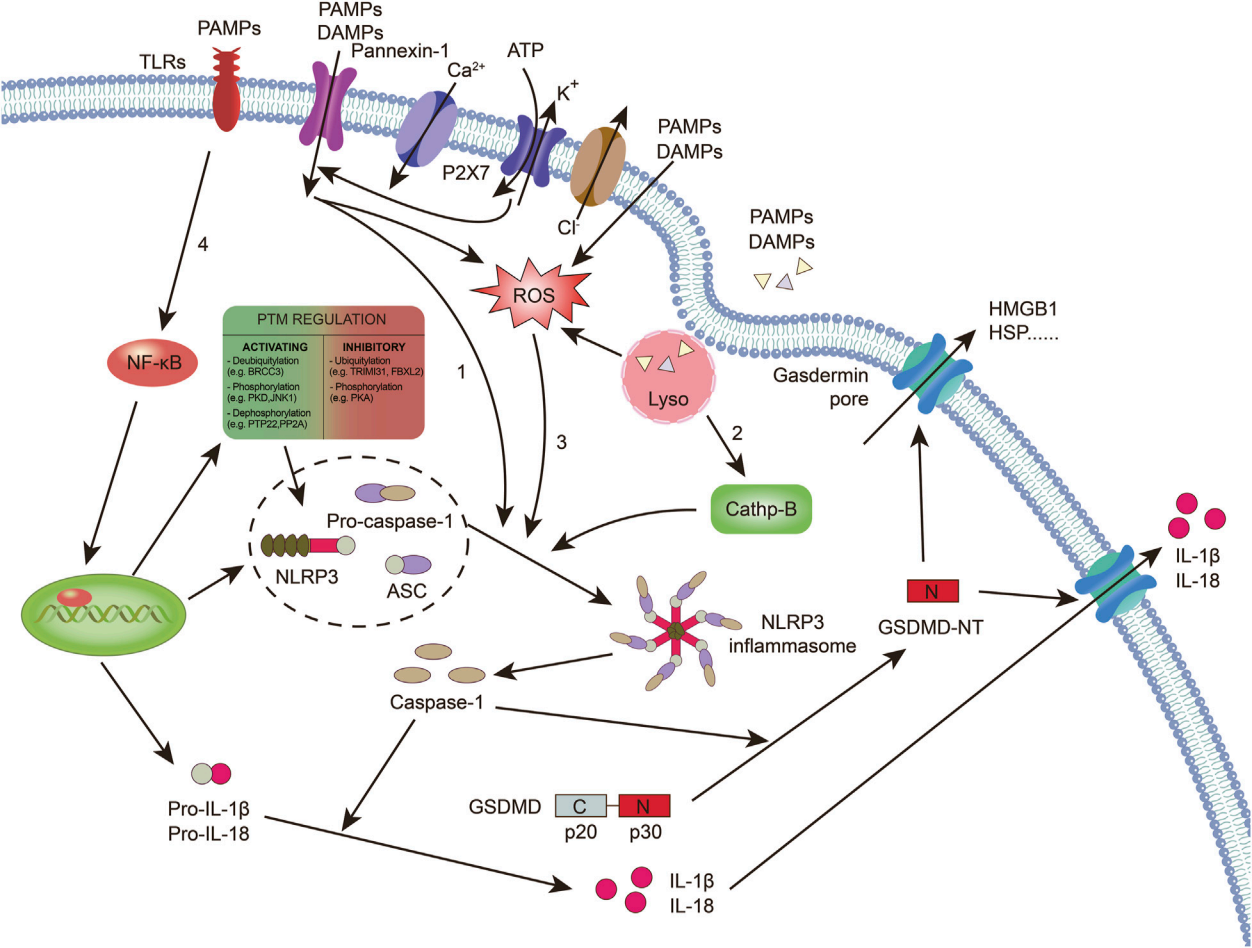
Caspase-1-dependent canonical pyroptotic cell death induced by NLRP3 inflammasome activation. The assembly of the NLRP3 inflammasome involves NLRP3 oligomerization and ASC recruitment, triggering the autocleavage of pro-caspase-1. This autocleavage leads to the activation of caspase-1, which converts inactive pro-IL-1β and pro-IL-18 into their bioactive and secreted forms, namely IL-1β and IL-18. Additionally, active caspase-1 cleaves GSDMD, generating GSDMD-NT, which forms pores on the plasma membrane, inducing pyroptosis. Various models have been proposed to explain the assembly of the NLRP3 inflammasome. 1): Extracellular ATP can activate the NLRP3 inflammasome through different mechanisms. This includes the activation of the P2X7 receptor leading to the opening of the pannexin-1 pore, allowing the entry of extracellular factors that directly interact with NLRP3. Alternatively, NLRP3 can sense either the efflux of K+ or the loss of membrane integrity. 2): Crystalline or particulate agonists can be phagocytosed, resulting in the release of lysosomal cathepsins B and L, which are detected by the NLRP3 inflammasome. 3): NLRP3 agonists such as DAMPs and PAMPs can trigger the production of ROS, which activates the NLRP3 inflammasome. It is important to note that these models are not mutually exclusive but rather interact with each other. 4): The activation of the NLRP3 inflammasome also requires “priming” with TLR agonists, such as LPS.. (ASC: apoptosis-associated speck-like protein; TLRs: toll-like receptors; DAMPs: damage-associated molecular patterns; GSDMD: gasdermin D; NT: n-terminal; HSP: heat shock proteins; HMGB-1: high mobility group box-1; IL: interleukin; NLRP3: NOD-like receptor protein 3; NF-κB: nuclear factor-κB; PAMPs: pathogen-associated molecular patterns; P2X7: purinergic receptor P2X, ligand-gated ion channel 7; ROS: reactive oxygen species).

The NLRP3 inflammasome is composed of several key components. The NLR protein in the NLRP3 inflammasome contains a conserved nucleotide-binding and oligomerization domain, C-terminal LRRs, and a pyrin domain (PYD) that facilitates multimerization (Xi et al., 2024). Upon activation of the NLRP3 inflammasome, the NLRs oligomerize through their nucleotide-binding and oligomerization domains (Fu and Wu, 2023). This leads to the recruitment of the adaptor protein apoptosis-associated speck-like protein (ASC) through PYD-PYD interactions (Fu and Wu, 2023). ASC then forms large speck-like structures and recruits pro-caspase-1 through caspase recruitment domain (CARD)-CARD interactions. Pro-caspase-1 undergoes autocatalytic cleavage, resulting in the formation of active caspase-1 p10/p20 tetramers (Ruan, 2019). These active caspase-1 tetramers mediate the maturation and secretion of IL-1β and IL-18 (Ruan, 2019; Fu and Wu, 2023). Additionally, caspase-1 can cleave GSDMD to generate GSDMD n-terminal (NT). GSDMD-NT forms plasma membrane pores, leading to the induction of pyroptosis (Lee et al., 2021; Nie et al., 2021). The canonical activation of inflammasomes is proposed to occur in two steps: priming and assembly (Bockstiegel et al., 2023). Priming involves the initial activation of toll-like receptors by agonists such as lipopolysaccharide (LPS) (Fu and Wu, 2023). This trigger signaling cascades, primarily through the nuclear factor-κB (NF-κB) pathway, which leads to the transcriptional upregulation of pro-inflammatory mediators like pro-IL-1β. Following priming, the activated inflammasome assembles in response to various PAMPs or DAMPs (Fu and Wu, 2023; Krantz et al., 2023). This assembly forms a large molecular platform that activates inflammatory caspases and processes pro-IL-1β (Huang et al., 2023). The activation of NLRP3 inflammasome requires “priming” with TLR agonists to initiate signaling cascades (primarily nuclear factor-κB (NF-κB)-dependent pathway) that ultimately promote a transcriptional response to upregulate pro-inflammatory mediators (Figure 1).

K^+^ efflux is recognized as a crucial upstream signal for NLRP3 inflammasome activation (Zhou et al., 2020). Activation of NLRP3 also requires the mobilization of Ca^2+^ (Diaz-Del-Olmo et al., 2021). Mobilization of Ca^2+^ occurs when extracellular Ca^2+^ moves across channels in the plasma membrane and the Ca^2+^ stored in the endoplasmic reticulum is released into the cytoplasm, which can induce NLRP3 inflammasome activation (Watanabe et al., 2020; Diaz-Del-Olmo et al., 2021). In addition, Cl^−^ was implicated in NLRP3 activation. Cl^−^ channel blockers and elevated levels of extracellular Cl^−^ can inhibit, whereas reduced levels of Cl^−^ can enhance, the activation of NLRP3 (Zhou et al., 2020). Cl^−^ efflux may be downstream of K^+^ efflux and affects ASC polymerization, whereas K^+^ efflux promotes NLRP3 oligomerization (Zhou et al., 2024).

In addition to the ion channels mentioned earlier that can activate the NLRP3 inflammasome, organelle dysfunction can also trigger inflammasome activation (Movahedpour et al., 2023). In a study, sterile lysosomal rupture caused by L-leucyl-L-leucine methyl ester is sufficient to trigger NLRP3 inflammasome activation, whereas suppression of phagosomal acidification or cathepsin B blocks NLRP3 activation (Movahedpour et al., 2023). Mitochondrial dysfunction contributes to the activation of the NLRP3 inflammasome (Kodi et al., 2024). When damaged organelles accumulate due to deficiencies in autophagic proteins, dysfunctional mitochondria release mitochondrial DNA, leading to NLRP3 inflammasome activation (Kodi et al., 2024). Additionally, mitochondrial ROS (mtROS) can initiate NLRP3 activation (Kodi et al., 2024). Inhibiting autophagy or mitophagy results in the buildup of mitochondrial ROS and subsequent NLRP3 activation (Chen et al., 2022; Lewisluján et al., 2022; Zhang et al., 2022; Lu et al., 2023).

Post-transcriptional modifications of the NLRP3 protein occur in unstimulated cells and during priming and activation to modulate its activation and function (Swanson et al., 2019). In peritoneal macrophages, tripartite motif 31 ubiquitylates NLRP3, targeting it for proteasomal degradation (Chan and Schroder, 2020). The E3 ubiquitin ligase f-box and leucine-rich repeat protein 2 are suggested to prevent NLRP3 activation by directing it to the proteasome in lung epithelial cells (Han et al., 2020). Additionally, membrane-associated ring-ch-type finger 7, another E3 ubiquitin ligase, inhibits NLRP3 downstream of dopamine-induced D1 receptor signaling (Sandall and Macdonald, 2019). Another regulatory level involves phosphorylation of NLRP3 at Ser291 (or Ser295 in humans) by protein kinase A, leading to K48- and K63-linked ubiquitination and subsequent proteasomal degradation (Ren et al., 2019). Song et al. Demonstrated that following LPS priming, NLRP3 is phosphorylated on Ser194 by c-Jun N-terminal kinase 1, promoting NLRP3 oligomerization upon activation by canonical stimuli (Spalinger et al., 2023). In monocytic cells, a study revealed that dephosphorylation of NLRP3 at Tyr861 by protein tyrosine phosphatase nonreceptor 22 induces NLRP3 activation (Spalinger et al., 2020). Whether these pathways synergize to tightly control NLRP3 activity remains elusive.

## 3 Mechanisms of pyroptosis

Recent advances have shed light on the molecular mechanisms of pyroptosis, a form of programmed cell death induced by the agonist of the NLRP3 inflammasome (McKee and Coll, 2020; Yuan et al., 2020; Chen et al., 2022; Zhong et al., 2023). Among these mechanisms, GSDMD has emerged as a crucial mediator of pyroptosis. GSDMD belongs to a family of proteins named GSDMs, which share a pore-forming domain (McKee and Coll, 2020). Cleavage of GSDMD by caspase-1 or caspase-4/5/11 releases GSDMD-NT, the NT domain of GSDMD (McKee and Coll, 2020). GSDMD-NT then forms these pores in the plasma membrane, leading to cell swelling and osmotic lysis (Ren et al., 2019). Other members of the GSDM family also possess pore-forming activity but are not targeted by inflammatory caspases (Chen et al., 2022). The cleavage of GSDMD occurs at a conserved residue called D276, resulting in the separation of GSDMD into two domains: GSDMD-NT (p30) and GSDMD C-terminal domain (p20) (Zhong et al., 2023). GSDMD-NT can interact with lipids in the plasma membrane and assemble into large oligomeric pores (Yuan et al., 2020). This disruption of the cell membrane integrity leads to an increase in intracellular osmotic pressure and the release of inflammatory intracellular contents, including high mobility group box-1 (HMGB-1) and heat shock protein (Yuan et al., 2020; Chan et al., 2023). This process, characterized by caspase-1-dependent cleavage of GSDMD, is known as the pyroptotic pathway (Chan et al., 2023).

## 4 Overview of exercise

**FIGURE 2:**
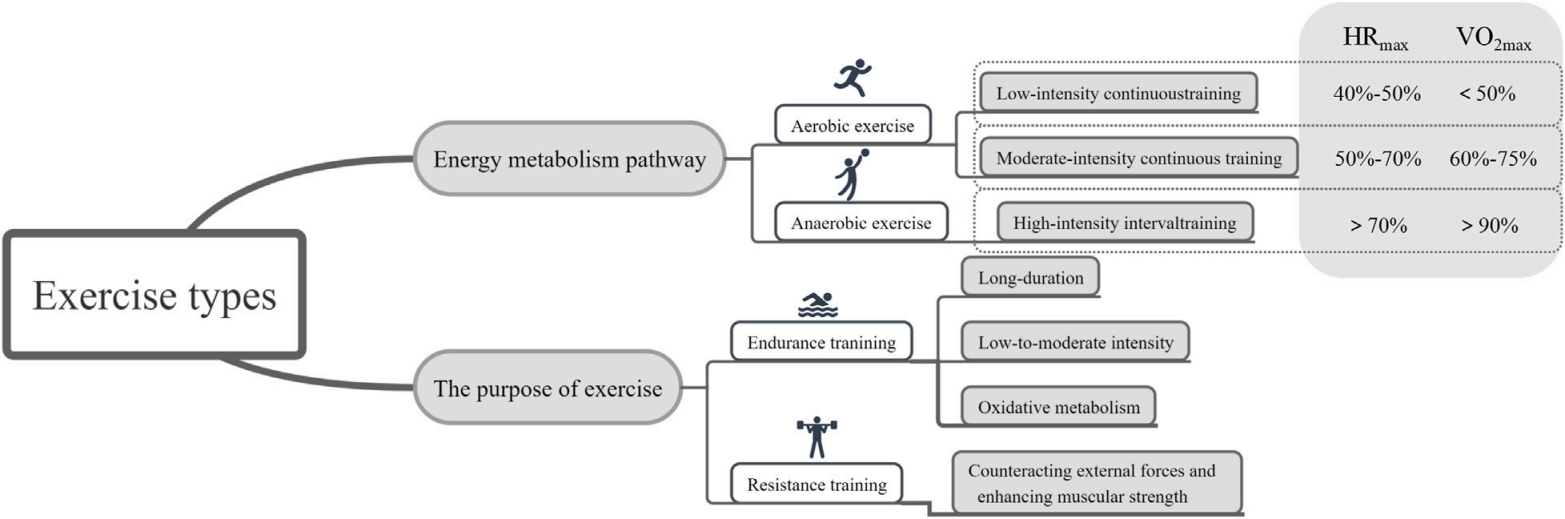
Regarding the specific classification of exercise and the criteria for its determination. (HR_max_: maximum heart rate; VO_2max_: maximum volume of oxygen).

Exercise refers to the movement of the body, typically involving the musculoskeletal system, to maintain health, improve physical fitness, promote cardiovascular health, and enhance the overall quality of life (Vella et al., 2017). It is a crucial component for maintaining both physical and mental wellbeing (Vella et al., 2017). By selecting forms of exercise that suit individual needs and goals, comprehensive health benefits can be achieved (Vella et al., 2017). The most common classification of exercise is based on the primary energy metabolism pathways, dividing it into aerobic and anaerobic exercise (Paluch et al., 2024) (Figure 2).

Aerobic exercise is a form of activity that generates energy through oxidative metabolism (Mueller et al., 2021). This type of exercise involves relatively low to moderate intensity over an extended period to ensure the body can supply enough oxygen to support energy production (Mueller et al., 2021). Aerobic exercise is characterized by lower intensity, safety, rhythmic, and sustained durations, with relatively minor stress on various organs, reducing the risk of exercise-related injuries (Mueller et al., 2021). Common aerobic exercise programs include low-intensity continuous training (LICT) and moderate-intensity continuous training (MICT) (Troosters et al., 2023). The intensity of exercise is typically measured using parameters such as maximum heart rate and maximum oxygen consumption (Blanks et al., 2019). For maximum heart rate (HR_max_), low-intensity exercise falls within the 40%–50% range, while moderate-intensity exercise falls within the 50%–70% HR_max_ range (Blanks et al., 2019). Regarding the maximum volume of oxygen (VO_2max_), values below 60% are considered low intensity, and those between 60% and 75% are considered moderate intensity (Blanks et al., 2019).

Anaerobic exercise involves high-intensity, momentary bursts of muscle activity in an “oxygen-deprived” state (Liao et al., 2022). It is characterized by high-intensity loads and brief durations, making it challenging to sustain for extended periods, and recovery from fatigue is slower (Liao et al., 2022). Anaerobic exercise enhances muscle strength, improves adaptability, and serves as a primary source of muscle growth (Casado et al., 2023). The intensity of anaerobic exercise is relatively high, and the sustainable duration is short, resulting in high-intensity loads that can lead to muscle fatigue and soreness (Casado et al., 2023). Common anaerobic exercise programs include resistance training (RT) and high-intensity interval training (HIIT) (May et al., 2020). Recently, HIIT has gained popularity as a time-efficient exercise strategy that has been proven to improve cardiovascular risk factors in various populations (May et al., 2020). This training method employs alternating patterns of work and rest to enhance cardiorespiratory endurance, promote fat burning, and provide more efficient training effects in a shorter time (May et al., 2020). High-intensity training is identified by an HR_max_ exceeding 70% or VO_2max_ exceeding 90% (Blanks et al., 2019).

Additionally, based on the primary training goals, exercise can be classified into endurance training (ET) and RT (Consitt et al., 2019). ET involves prolonged, continuous activities at relatively low loads, primarily relying on aerobic metabolism to produce energy (Rothschild and Bishop, 2020). It induces adaptations in the cardiovascular and musculoskeletal systems, supporting overall improvements in exercise capacity and performance (Miko et al., 2020). However, for older individuals who are overweight or obese, especially those with symptoms of osteoarthritis, regular ET may be uncomfortable and painful, necessitating the introduction of alternative forms of exercise (Cutrufello et al., 2020).

RT, on the other hand, offers various health benefits, supports body weight, and avoids imposing impact stress on joints (Schoenfeld et al., 2016). Therefore, RT may be an appealing exercise modality for overweight/obese older individuals. This type of training emphasizes resistance against external forces to enhance muscle strength, endurance, and mass, with each effort specifically targeting the resistance generated, designed to increase muscle strength and explosiveness (Carvalho et al., 2022).

Although most literature suggests that exercise can improve CVD, excessive endurance exercise (EEE) may have many potential adverse effects on cardiac structure and function (O'Keefe et al., 2012). Acutely, EEE can increase myocardial injury markers, lead to chamber dilation, and decrease right ventricular function (Levine, 2014). Chronically, concerns exist that this degree of EEE may lead to detrimental cardiac remodeling and fibrosis, as well as non-lethal arrhythmias, especially an increased risk of atrial fibrillation, and potentially more lethal ventricular arrhythmias (Nath et al., 2023). Recent studies also indicate that despite a more favorable overall profile of coronary heart disease risk in long-distance runners, they may have higher levels of atherosclerosis and coronary heart disease risk (Neumann et al., 2022; O'Keefe et al., 2021). However, the benefits of aerobic exercise on CVD mortality appear to be significantly diminished when running exceeds 30 miles per week or walking exceeds 46 miles per week (Schwartz et al., 2014). Even though that lack of physical activity is more prevalent than EEE in the overall population, the potential adverse effects may be more serious on a societal level for overall health and cardiovascular health (McCullough and Lavie, 2014). These studies also suggest that more is not necessarily better, and even low doses of aerobic exercise, particularly running, seem to confer benefits for long-term cardiovascular health and lifespan.

## 5 Mechanisms by which exercise regulates NLRP3 inflammasome activation

Exercise has long been acknowledged as an effective intervention in regulating the innate immune response (Pope and Wood, 2020). In general, LICT and MTCT have positive effects on the immune system, whereas HIIT tends to have the opposite effect (Xian et al., 2022). However, there is limited research on how exercise specifically influences the activity of the NLRP3 inflammasome.

From the perspective of material metabolism, many studies have reported that glucose and lipids can directly activate the NLRP3 inflammasome (Vandanmagsar et al., 2011; Zhang et al., 2021; Baik et al., 2023), while exercise can directly alleviate glucose and lipid metabolism, reducing the levels of blood sugar and lipids, thereby improving CVD. There is currently no literature reporting that exercise can directly regulate the activation pathway of NLRP3 inflammasome through glucose and lipid metabolism (Mardare et al., 2016; Ma et al., 2021). Therefore, it boldly speculates that the effect of exercise on NLRP3 inflammasome activation is likely to be exerted through the regulation of glucose and lipid metabolism.

From the mitochondrial standpoint, since most studies suggest that mitochondria are involved in regulating NLRP3 inflammasome activation (Chen et al., 2019; Hong et al., 2021), it is speculated that exercise adaptation of mitochondria may affect the NLRP3 inflammasome activity. Earlier studies demonstrated that MICT significantly decreased the expression of inflammatory cytokines TNF-α, IL-6, and monocyte chemoattractant protein-1 induced by metabolic disorders, coupled with an upregulation in mitochondrial proteins (Liu et al., 2017; Chen et al., 2018). Conversely, HIIT led to mitochondrial dysfunction and an augmentation in the secretion of pro-inflammatory factors (Memme et al., 2021). Moreover, additional investigations indicated that MICT facilitated mitochondrial biogenesis, bolstered antioxidant capacity, and restrained the overactivation of the NLRP3 inflammasome (Mason et al., 2020; Powers et al., 2020). Furthermore, aerobic exercise mitigated cardiac dysfunction by modulating the expression of proteins implicated in mitochondrial quality and NLRP3/caspase-1/IL-1β signaling (Salo et al., 1991). In pathological conditions, mitochondrial damage leads to increased production of mtROS (Chen et al., 2020). The elevated mtROS mediates the activation of the NLRP3 inflammasome through thioredoxin interacting protein (TXNIP) and thioredoxin (Zhang et al., 2021). These findings imply that MICT might diminish mitochondrial ROS production through the regulation of mitochondrial quality control, enhancement of mitochondrial function, and the facilitation of damaged mitochondria clearance. Consequently, this could inhibit the NLRP3 inflammasome pathway and alleviate exaggerated inflammatory responses.

From molecular mechanisms in the cell, the key factors in exercise-mediated improvement of CVD through the NLRP3 inflammasome may be related to the regulation of the NF-κB signaling pathway. Some studies have focused on the NF-κB pathway as a critical node to explore the molecular mechanisms among exercise and NLRP3 inflammasome (Wang et al., 2019; Zhou et al., 2022). For example, research has demonstrated that aerobic exercise is capable of reducing the expression of nicotinamide adenine dinucleotide phosphate oxidase 4 (NOX4), ROS, TNF-α, IL-18, NF-κB p65, and the NLRP3 inflammasome (Zhou et al., 2022). These findings suggest that exercise may ameliorate the pathological alterations in diabetes mellitus through the modulation of the NOX4/ROS/NF-κB/NLRP3 inflammasome signaling cascade (Zhou et al., 2022). In a separate study, Wang et al., 2019 observed that aerobic exercise suppresses the acetylation of forkhead box transcription factor O1 (FOXO1) in the brain tissue of diabetic rats, which in turn promotes the phosphorylation of FOXO1, thus inhibiting expression of NF-κB and NLRP3 inflammasome protein. This downregulation contributes to the inhibition of inflammatory responses, indicating that exercise may exert anti-inflammatory effects via the FOXO1/NF-κB/NLRP3 inflammasome pathway (Wang et al., 2019). Although studies have shown that exercise improves CVD through the NLRP3 inflammasome (Li et al., 2021; Zhou et al., 2022), further research is needed to elucidate the detailed molecular mechanisms by which exercise regulates the NLRP3 inflammasome.

## 6 Exercise, NLRP3 inflammasome, and CVD

### 6.1 Exercise improves AS and inhibits NLRP3 inflammasome

The pathogenesis of AS involves multiple processes, including endothelial dysfunction, low-density lipoprotein accumulation and oxidation, monocyte and lymphocyte recruitment, smooth muscle cell migration and proliferation, proinflammatory cytokine activation, and platelet adhesion (Zeng et al., 2019). Notably, the augmented release of inflammatory cytokines primarily attributed to endothelial cells, macrophages, and smooth muscle cell pyroptosis or NLRP3 inflammasome activation significantly contributes to AS formation and development (Zhaolin et al., 2019). Specifically, NLRP3 inflammasome-dependent pyroptosis triggers endothelial dysfunction, thereby acting as a catalyst for AS in these cells (Sun et al., 2017). This highlights the crucial role of NLRP3 inflammasome in promoting the release of inflammatory mediators and contributing to the pathological changes associated with AS.

Exercise can reduce the inflammatory death of local endothelial cells, slowing the development of AS plaques (Xu et al., 2019). A study found that voluntary wheel running, a natural type of aerobic exercise in the murine model could decrease the protein levels of the inflammasome components and markedly inhibit the caspase-1 activity in endothelial cells within the aorta of mice fed with a high-fat diet (HFD) (Lee et al., 2020). Recently, another study has demonstrated that exercise-induced a significant downregulation of m6A modification and methyltransferase-like 14 (Yang et al., 2023). This protein binds to the m6A sites of nuclear paraspeckle assembly transcript 1 (NEAT1) and promotes NEAT1 expression through subsequent YT521-B homology domain-containing 1, which transcriptionally promotes NLRP3 expression and endothelial pyroptosis (Yang et al., 2023). As a result, exercise effectively inhibits NLRP3 expression and endothelial pyroptosis, preventing AS plaque formation (Yang et al., 2023). In addition, it is widely recognized that fibroblast growth factor 21 (FGF21), a well-established negative risk factor for AS expressed in the aorta, exerts inhibitory effects on NLRP3 inflammasome activity, thereby attenuating aortic pyroptosis to prevent AS development (Zeng et al., 2020). Interestingly, aerobic exercise increases FGF21 sensitivity while downregulating the expression of pyroptosis-related proteins mediated by NLRP3 inflammasome in the aorta (Li et al., 2022). These findings suggest that activated FGF21 may be involved in aerobic exercise inhibiting NLRP3 inflammasome-mediated pyroptosis in the aorta. However, the current study has not investigated whether exercise improved AS via inhibiting NLRP3 inflammasome and pyroptosis in these cells, which needs further research in the future.

### 6.2 Exercise improves HHcy and inhibits NLRP3 inflammasome

Homocysteine (Hcy), a non-essential amino acid sulfur, is derived from methionine and is used for methylation of DNA/RNA methylation (Veeranki and Tyagi, 2013). HHcy refers to a condition where plasma Hcy levels exceed 15 μmol/L and has been associated with various diseases especially AS (Winchester et al., 2014). HHcy promotes the generation of ROS through mechanisms such as mixed disulfide formation and auto-oxidation (Veeranki and Tyagi, 2013; Winchester et al., 2014). Recent research has demonstrated that HHcy-induced activation of the NLRP3 inflammasome in macrophages contributes to vascular inflammation and AS by activating caspase-1-mediated pyroptosis (Wang et al., 2017). In addition, acid sphingomyelinase upregulation by Hcy promotes clustering of lipid rafts mediating the assembly of NADPH oxidase complex resulting in increased ROS generation followed by NLRP3 inflammasome activation leading to pyroptosis contributing towards the development of AS (Liu et al., 2022).

Although direct evidence is currently lacking regarding whether exercise specifically modulates NLRP3 inflammasome activation or pyroptosis about HHcy, it has been observed that exercise can lower Hcy levels (Liu et al., 2022). In a folate-restricted model of HHcy, exercise can reduce the increase in plasma Hcy levels by increasing betaine Hcy s-methyltransferase levels in the kidneys and promoting nonclassical remethylation to convert Hcy into methionine (Vincent et al., 2006; Neuman et al., 2013). Moreover, apart from reducing plasma Hcy levels, exercise holds the potential for mitigating Hcy-induced lipid peroxidation and ameliorating reductions in superoxide dismutase and catalase activity, both of which are implicated in the progression of AS (Neuman et al., 2013). These works suggest that exercise might also inhibit NLRP3 inflammasome activation or pyroptosis as a part of its overall impact on reducing HHcy-related complications. Given these findings, further investigation is warranted to explore whether exercise precisely influences NLRP3 inflammasome activation or pyroptosis and their role in HHcy management. Understanding this relationship could provide valuable insights into developing targeted interventions for individuals with elevated Hcy levels and associated CVD.

### 6.3 Exercise improves obesity and inhibits NLRP3 inflammasome

Obesity, commonly associated with chronic low-grade inflammation, is characterized by the pathological enlargement of adipose tissue (AT) primarily due to excess energy accumulation as fat (Spalding et al., 2008; Wada et al., 2017). Mice with HFD-induced obesity exhibit increased expression of caspase-1, ASC, and NLRP3. However, knocking out the *Nlrp3* or *Caspase-1* gene suppresses obesity-induced fat depot (Stienstra et al., 2011). Therefore, targeting the activation of NLRP3 inflammasome or pyroptosis represents a promising approach for improving obesity.

A study found that exercise decreased protein expression of inflammasome components (NLRP3 and caspase-1) in bone marrow-derived macrophages (BMDM) and AT isolated from mice with diet-induced obesity (Javaid et al., 2021). Another study investigated perform mice fed either a standard diet or an HFD and subjected to regular ET or RT and discovered that RT attenuated increased NLRP3 expression and reduced levels of IL-18 in isolated AT, while ET effectively reduced the expression of TNF-α and IL-18 in supernatant from AT, suggesting that exercise can reduce inflammasome activation in ATs and achieve systemic downregulation of inflammatory cytokines (Wada et al., 2017). Besides AT, endothelial dysfunction emerges early on in CVD associated with obesity (Stienstra et al., 2011; Wada et al., 2017). A study demonstrated that engaging in voluntary running while on an HFD led to a significant reduction in active caspase-1 levels within the endothelial cells lining the aorta when compared to sedentary mice on the same diet (Li et al., 2023). These findings indicate that voluntary running alleviates impaired blood vessel function via inhibiting NLRP3 inflammasome activation (Li et al., 2023). In addition to endothelial cells, exercise suppressed NLRP3 inflammasome activation as revealed by downregulated IL-1β and IL-18 in BMDM (Li et al., 2023). This body of research demonstrates that exercise exerts inhibitory effects on NLRP3 inflammasome activation across various cell types to ameliorate obesity.

It’s worth noting that a human study found that exercise reduces plasma IL-18 levels in obese individuals, indirectly suggesting the inhibition of the NLRP3 pathway through exercise to improve obesity (Stienstra et al., 2011). Furthermore, 8-week high-intensity and aerobic interval training (three times/week) in men and women with metabolic syndrome resulted in decreased IL-18 mRNA levels in abdominal AT and a numerical decrease in plasma IL-18 concentration (Stienstra et al., 2011; Stienstra et al., 2011; Stienstra et al., 2011; Stienstra et al., 2011). Similarly, a pilot study, conducted among thirty-seven obese individuals demonstrates that exercise intervention, primarily consisting of activities such as walking, jogging, and functional exercise circuits designed to enhance aerobic capacity and speed, leads to significant reductions in ASC mRNA expression levels when compared to the hypocaloric group without any form of exercise participation (Barrón et al., 2020). These findings indicate an inverse relationship between ASC mRNA expression and aerobic interventions. Another randomized controlled trial involving 36 obese inactive subjects further delineated the type of exercise intervention and demonstrated both HIIT and MICT significantly reduced NLRP3 gene expression in serum samples from all subjects, strongly suggesting that diversity intensity interval training can inhibit NLRP inflammasome in obese (Armannia et al., 2022).

Therefore, exercise is considered a crucial strategy for reducing inflammation and metabolic disorders associated with obesity. Further research will enhance understanding of the complex relationship between exercise and NLRP3 inflammasome, leading to more effective interventions for managing obesity-related diseases.

### 6.4 Exercise improves diabetes and inhibits NLRP3 inflammasome

Diabetes is a metabolic disease that poses a significant threat to human health. Type 2 diabetes, a significant risk factor for both microvascular and macrovascular diseases, accounts for 90% of diabetes cases and is a leading cause of death, particularly due to coronary heart disease (Einarson et al., 2018). Inflammatory processes play a crucial role in the development of complications associated with diabetes (Roncero-Ramos et al., 2018). Recently, NLRP3 inflammasome and pyroptosis have emerged as key contributors to insulin resistance (IR) (Roncero-Ramos et al., 2018). Peripheral blood-derived macrophages from drug-naïve patients with type 2 diabetes show increased expression of NLRP3 and ASC along with caspase-1 activation and IL-1β maturation (Lee et al., 2013). *In vivo* and *in vitro* studies have reported that high glucose stimulates IL-1β secretion in pancreatic β cells, resulting in their death through the activation of NLRP3 inflammasome (Zhou et al., 2010; Wu et al., 2019). The various modes of activating the NLRP3 inflammasome are fundamental factors influencing its complex effects on the progression of type 2 diabetes (Chen et al., 2021).

Engaging in aerobic exercise can improve IR and reduce the expression of NLRP3 and IL-1β in individuals with type 2 diabetes in aortic tissue, suggesting its positive impact on IR by inhibiting NLRP3 inflammasome (Hassanpour Soleimani et al., 2021). HMGB1 is a major pro-inflammatory cytokine released as a result of pyroptosis (Biguetti et al., 2019). This work also found that exercise can reduce the secretion of HMGB1, which is significantly increased in individuals with diabetes and contributes to disease progression (Hassanpour Soleimani et al., 2021). Previous studies have shown that exercise has the potential to lower HMGB-1 levels in circulation and tissues of diabetic patients, possibly by inhibiting NLRP3 inflammasome activation and pyroptosis (Haß et al., 2022; Haß et al., 2023). Furthermore, exercise decreased circulating levels of IL-1, which may potentially protect against IL-1-mediated destruction of β-cells (Stumvoll et al., 2005). Although there is currently no direct evidence on whether aerobic exercise specifically affects NLRP3 inflammasome activation or pyroptosis and thus decreases levels of blood glucose, these findings highlight the significant role played by exercise in regulating IR through its impact on NLRP3 inflammasome and pyroptosis.

### 6.5 Exercise improves DCM and inhibits NLRP3 inflammasome

DCM is a distinct cardiac phenotype observed in diabetic patients characterized by structural changes such as cardiac hypertrophy, cardiomyocyte death, and fibrosis, as well as functional abnormalities (Sun et al., 2021). The molecular mechanisms underlying DCM involve various factors including hyperglycemia, IR, fatty acids, oxidative stress, mitochondrial dysfunction, inflammation, and endothelial dysfunction (Chen et al., 2020). In particular, inflammation is believed to be present in the early stages of diabetes and plays a crucial role in promoting DCM (Luo et al., 2017). Emerging evidence has verified that NLRP3 inflammasome-mediated cardiomyocyte pyroptosis is a key participant in DCM (Xie et al., 2020). Human diabetic hearts also exhibit elevated activation of the NLRP3 inflammasome and cardiac pyroptosis compared to non-diabetic heart tissues (Zhang et al., 2015; Xie et al., 2020). Moreover, high glucose levels (35 mM) significantly induce increased protein expression of NLRP3, caspase-1, and IL-1β accompanied by noticeable cardiomyocyte pyroptosis, leading to loss of contractile units and cardiac dysfunction (Zhang et al., 2015). In contrast, silencing the *Nlrp3* gene ameliorates cardiac inflammation and pyroptosis and improves cardiac function by ameliorating cardiac inflammation and pyroptosis both *in vivo* and *in vitro* experiments (Chen et al., 2020).

Exercise intervention has been shown to effectively prevent and treat DCM by modulating the NLRP3 inflammasome. For example, the expressions of NLRP3, caspase-1-p20, caspase-1p20/caspase-1, and IL-1β were increased in the myocardium of HFD-induced obese mice (Lee et al., 2018). However, treadmill exercise inhibited these parameters (Sun et al., 2021). This demonstrates that exercise training can prevent obesity-induced cardiac inflammasome formation, pyroptosis activation, and pro-inflammatory response (Sun et al., 2021). In addition, recent studies in DCM mice have demonstrated that although exercise has a limited impact on interstitial fibrosis, it can effectively reverse cardiac dysfunction by reducing the activity of NLRP3 inflammasome and inhibiting pyroptosis (Takahashi, 2019; Zhang et al., 2019). Moreover, in DCM rat models, elevated expression levels of the P2X7 receptor, NLRP3, caspase-1, and serum IL-1β were observed in the myocardium (Wang et al., 2022). However, following a 12-week treadmill running regimen in these rats, improvements were observed in terms of collagen deposition, cell disorder, as well as the expression levels of NLRP3, caspase-1, P2X7 receptor, and IL-1β within their heart (Zhang et al., 2015). Similarly, aerobic exercise also can inhibit the thioredoxin interacting protein (TXNIP)/NLRP3 inflammasome pathway and alleviate endothelial dysfunction in atherosclerotic coronary arterioles (Hong et al., 2018). These findings suggest that aerobic exercise can effectively mitigate fibrosis in the hearts subjected to an HFD and inhibit the activation of the NLRP3 inflammasome and pyroptosis in the myocardium. The effectiveness of exercise intervention on the NLRP3 inflammasome depends on the duration and intensity of exercise (Hong et al., 2018; Khakroo et al., 2019). Chronic exercise with moderate intensity significantly decreases the expression of the NLRP3 gene and levels of IL-1β, and IL-18 cytokines in serum (Khakroo et al., 2019). Conversely, chronic exercise with high intensity leads to a significant increase in gene expression of NLRP3 and levels of IL-1β, and IL-18 cytokines in serum (Khakroo et al., 2019). Therefore, personalized exercise regimens are necessary as there are currently no available guidelines; further research is needed.

### 6.6 Exercise improves hypertension and inhibits NLRP3 inflammasome

Hypertension is a potentially fatal yet preventable risk factor for CVD and accounts for the majority of cardiovascular mortality (Deussen and Kopaliani, 2023). Persistent inflammation plays a pivotal role in hypertension development, with activation of the inflammasome and pyroptosis being potential contributors to its onset (De et al., 2021). NLRP3 inflammasome activities have been implicated in various cell types associated with pulmonary hypertension, including pulmonary arterial smooth muscle cells, pulmonary artery endothelial cells, and systemic hypertension (Haß et al., 2023). Additionally, higher serum levels of IL-1β were observed in patients with hypertension compared to normotensive controls (Wu et al., 2022).

Exercise is commonly suggested as a lifestyle adjustment for individuals with hypertension due to various factors including inhibition of inflammation (Newman and Verdin, 2014; Chakraborty et al., 2018; Kong et al., 2021). β-Hydroxybutyrate (β-OHB) ester is primarily synthesized in the liver and transported to extrahepatic tissues, traditionally recognized as a crucial metabolic fuel during starvation periods (Newman and Verdin, 2014). Contemporary evidence indicates that ketone bodies like β-OHB can maintain physiological homeostasis by inhibiting NLRP3-inflammasome-mediated inflammation (Kong et al., 2021). Recently, a non-targeted metabolomics approach revealed nutritional intervention with β-OHB reversed the high salt-induced adverse effects including renal NLRP3-mediated inflammation, fibrosis, and hypertension (Chakraborty et al., 2018). Interestingly, exercise is associated with increased circulating levels of β-OHB (Luo et al., 2021). Therefore, exercise may raise β-OHB levels and subsequently inhibit renal NLRP3 inflammasome activation, thereby alleviating hypertension and preserving kidney function. Another *in vivo* study provided direct evidence of the impact of exercise training on the downregulation of NF-κB and NLRP3 pathways in the mesenteric artery of spontaneously hypertensive rats (SHR) (Luo et al., 2021), which revealed that three intensity training intensities from low to high significantly inhibited the expression of NLRP3 inflammasome component and NF-κB within the mesenteric artery and alleviate BP in SHR rat (Luo et al., 2021). In addition to the SHR rat model, Bal et al., 2022 discovered that regulated aerobic exercise also effectively reduces BP and suppresses protein expression of NLRP3, IL-1β, and caspase-1 in the heart within the deoxycorticosterone-acetate salt hypertension model. These findings from the diversity hypertension model demonstrate that exercise training effectively attenuates NLRP3 inflammasome activity, highlighting its potential as a therapeutic intervention for hypertension.

### 6.7 Exercise improves MI and inhibits NLRP3 inflammasome

MI, a common condition, refers to the death of heart muscle cells caused by a blockage in the coronary arteries (Wang et al., 2020). Following a heart attack, inflammatory reactions occur, characterized by the release of inflammatory cells, cytokines, and chemical hormones (Mezzaroma et al., 2011; Wang et al., 2020). These processes contribute to cardiac dysfunction, damage to the heart muscle, and remodeling (Mezzaroma et al., 2011; Wang et al., 2020). Indeed, NLRP3 inflammasome and pyroptosis-mediated inflammation have been reported to contribute to MI progression (Sandanger et al., 2013). Sandanger et al., 2013 first showed an increase in NLRP3 inflammasome activity in the left ventricle of the heart after MI. Furthermore, it appears that interfering with NLRP3 inflammasome signaling can prevent and mitigate damage caused by MI (Sandanger et al., 2013).

The research has shown that exercise training can delay the onset of ischemic reperfusion injury and MI (Thomas et al., 2016). In other words, exercise acts like ischemia preconditioning, which stimulates beneficial cellular responses. Azam Ahmadi et al., 2022 found HIIT can effectively decrease heart injury and NLRP3 expression in rats with MI. Interestingly, dynamin-related protein 1 (Drp1), an essential mitochondrial fission protein, can activate NLRP3 inflammasome through ROS generation after MI (Ahmadi et al., 2022; Jiang et al., 2022). However, the Azam Ahmadi team’s work demonstrates exercise preconditioning (EP) in a short period did not affect Drp1 (Ahmadi et al., 2022), while inhibiting NLRP3 expression. This finding indicates that EP in a short period is required to inhibit NLRP3 inflammasome independently of Drp1; the reduction of NLRP3 can occur through other mechanisms (Jiang et al., 2022).

### 6.8 Exercise affects HF and regulates NLRP3 inflammasome

HF means that the heart is unable to pump sufficiently to sustain blood flow to meet the needs of the body (Sacco et al., 2014). It represents the advanced stage of various CVD, including myocarditis, MI, cardiomyopathy, hypertension, atrial fibrillation, valvular heart disease, alcohol abuse, and infection (Bracey et al., 2013). Recent studies on calcineurin transgene mice have shown elevated mRNA of NLRP3 and enhanced cleavage of caspase-1 along with cardiac hypertrophy and ventricular dilatation (Bracey et al., 2013; Napodano et al., 2023). Genetic ablation of NLRP3 or administration of IL-1 receptor antagonist has been found to attenuate cardiac inflammation and systolic dysfunction (Bracey et al., 2013; Zhang et al., 2014; Napodano et al., 2023). Additionally, activation of NLRP3 inflammasome has been observed in LPS-stimulated cardiac fibroblasts and myofibroblasts, suggesting that its potential contribution to myocardial dysfunction through NLRP3 inflammasome or pyroptosis (Zhang et al., 2014; Boza et al., 2016). Although more research is needed to fully understand how the NLRP3 inflammasome or pyroptosis contributes to HF pathogenesis and progression; recent studies indicate that targeting this pathway could lead to new treatments for this debilitating condition (Lu et al., 2022; Xin et al., 2022).

Studies have shown that EP can regulate the NLRP3 inflammasome, reducing downstream inflammatory cytokines and protecting the heart (Li et al., 2020; Ma et al., 2021; Zhang et al., 2022). Li et al., 2020 found that moderate-intensity EP is more effective in protecting cardiac function and inhibiting the expression of TXNIP, NLRP3, NF-ĸB p65, and caspase-1 in the heart. As expected with moderate-intensity exercise, 8 weeks of aerobic exercise at this level inhibited protein expression related to myocardial NLRP3/caspase-1/IL-1β signaling pathways in mice with myocardial hypertrophy (Ma et al., 2021). In addition, Sandanger et al., 2013 show that the NLRP3 inflammasome is upregulated in myocardial fibroblasts post-MI, and maybe a significant contributor to infarct size development during ischemia-reperfusion. These findings highlight the potential therapeutic value of moderate-intensity exercise for cardiovascular health. However, exhaustive exercise (EE), characterized by sustained high-intensity exercise commonly practiced by athletes and soldiers alike, may lead to adverse reactions such as myocardial inflammation. It is reported that EE can increase NLRP3 expression and induce cardiac dysfunction which can be mitigated by EP at different intensities (Zhang et al., 2015). Importantly, moderate-intensity EP has cardioprotective effects on ventricular systolic and diastolic functions, indicating its superior efficacy compared to other intensities (Zhang et al., 2014).

Importantly, a clinical trial with 54 HF patients showed that exercise increased ASC methylation, decreased IL-1β and ASC mRNA levels compared to the control group in plasma, suggesting that exercise may improve HF via epigenetic regulation of ASC (Butts et al., 2018). Recently, our research team has reported for the first time that NLRP3 inflammasome-mediated pyroptosis contributes to the pathogenesis of non-ischemic dilated cardiomyopathy in cellular, murine, and human models (Zeng et al., 2020). Furthermore, numerous studies have investigated exercise intervention in patients with dilated cardiomyopathy and have demonstrated its safety and efficacy in improving exercise capacity and quality of life among these individuals (Stolen et al., 2003; Beer et al., 2008). Consequently, it is meaningful to investigate whether exercise can exert inhibitory effects on NLRP3 inflammasome activation and subsequent pyroptotic cell death in dilated cardiomyopathy. Preliminary data suggest that exercise may effectively inhibit NLRP3 inflammasome activation in dilated cardiomyopathy (unpublished data).

Although these experiments indicate that exercise improves HF by inhibiting NLRP3 inflammasome activation, there is still debate (Butts et al., 2018). For example, previous work reported that exercise training consisting of three sessions per week lasting for 60 min each, including aerobic exercises, strength exercises targeting major muscle groups, and stretching exercises have no effect on serum levels of pro-inflammatory cytokines (TNF-α, IL-1β, IL-8, and monocyte chemoattractant protein-1) of patients with severe Chagas cardiomyopathy (Rodrigues Junior et al., 2020), suggesting that exercise training may benefit patients with severe Chagas cardiomyopathy independent of its impact on inflammasome (Rodrigues Junior et al., 2020).

Most studies suggest that exercise improves HF by inhibiting the activation of NLRP3 inflammasomes. Nevertheless, a minority of research suggests that engaging in activities like EE might potentially worsen the progression of HF. It is imperative for future investigations to thoroughly examine the role played by NLRP3 inflammasome activation in exercise-induced exacerbation of HF.

## 7 Summary and prospects

**FIGURE 3:**
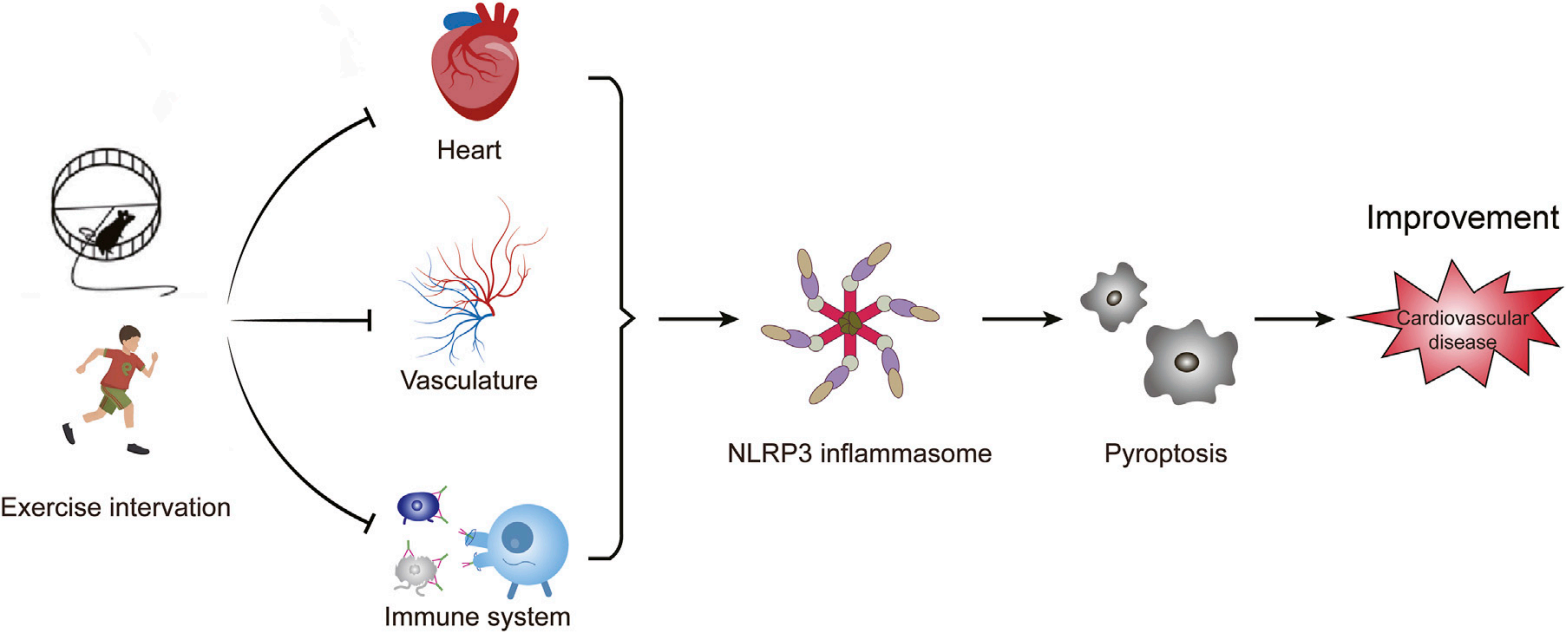
Exercise intervention affects cardiovascular disease by NLRP3 inflammasome-mediated pyroptosis. The activation of NLRP3 inflammasome induces pyroptosis, which promotes vascular inflammation and heart remodeling, and ultimately leads to cardiovascular diseases. Exercise affects the progression of cardiovascular diseases by inhibiting the activation of the NLRP3 inflammasome.

Exercise plays an important regulatory role in the development of various CVD by influencing the activation of the NLRP3 inflammasome (Figure 3). This impact has been observed in different types of cells and tissues, both *in vivo* and *in vitro* models of CVD (Table 1). This review highlights the close relationship between aerobic exercise, NLRP3 inflammasome, and CVD. It suggests that aerobic exercise can alleviate pyroptosis and improve cardiovascular-related diseases by modulating the NLRP3 inflammatory signaling pathway. Different patterns of exercise have varying impacts on the NLRP3 inflammasome; therefore, further research is needed to determine their optimal effects on specific types of cells and their underlying molecular mechanisms involving the NLRP3 inflammasome. Currently, there is a greater emphasis on animal experiments investigating the influence of exercise on NLRP3 inflammasome or cell pyroptosis while human studies are limited with small sample sizes in this area. Conducting comprehensive research on humans would provide a scientifically sound basis for understanding how exercise regulates NLRP3 inflammasome activity and promotes overall health. Hence, there is a need for methodologically rigorous large-scale human studies to determine ideal patterns of exercise.

**TABLE 1:** Types of cells and tissue injury subjected to NLRP3 inflammasome by exercise intervention in various CVD.

Diseases	Exercise type	Specific exercise intervention	Cell types/Organ	Injury types	Effects	References
AS	Aerobic exercise	Long-distance walking for at least 60 min at least 5 days a week	Human plasma*		NLRP3 inflammasome inactivation	Yang et al. (2023)
AS	Aerobic exercise	Run on a motorized rodent treadmill 5 days a week	Mouse plasma, heart, and aortic endothelial cells	HFD	NLRP3 inflammasome inactivation	(Yang et al., 2023)
AS	Aerobic exercise	Treadmill exercise training for 12 weeks	Serum, the aorta, and the right lobe of the liver	HFD	FGF21 and NLRP3 inflammasome-mediated pyroptosis	Li et al. (2022)
HHcy	Aerobic exercise	Wheel-exercised mice had free access to their wheels 24 h/d for 5 days/wk	Plasma, liver tissue, and kidney	AIN-93G semi-purified diet	Exercise reduced circulating Hcy, which may affect NLRP3 inflammasome-mediated pyroptosis	Neuman et al. (2013)
Obesity	Endurance training or resistance training	at 0.15 m/s, increasing every 3 min by 0.05 m/s; for 5 times/week for 3 min and 3 series	Mouse adipose tissue and serum	HFD	NLRP3 expression, levels of TNF-α and IL-18	Mardare et al. (2016)
Obesity	Aerobic exercise	Treadmill exercise for 8 weeks	Adipose tissue and bone marrow-derived macrophages	HFD	NLRP3 inflammasome	Javaid et al. (2021)
Obesity	Aerobic exercise	Running wheel access	Endothelial cells of the aorta	HFD	Activation of NLRP3 inflammasome	Lee et al. (2013)
Obesity	Aerobic exercise and resistance training	High-intensity aerobic interval training and strength training	Human serum*		IL-18, IL-6, and TNF-α	Stensvold et al. (2012)
Obesity	Aerobic exercise	12 weeks of intervention with exercise	Human serum*	4 subjects with metabolic syndrome	IL-18	(Troseid et al., 2009)
Obesity	Aerobic exercise	Hypocaloric diet exercise	Human peripheral blood*		ASC gene expression and IL-1β	Barrón et al. (2020)
Obesity	Aerobic exercise	High-intensity interval and moderate-intensity continuous training	Human serum samples*		The expression of NLRP3	Armannia et al. (2022)
Diabetes	Aerobic exercise	A treadmill for 6 weeks, 5 sessions per week	Rats adipose tissue and aortic tissue sampling	Streptozotocin	HMGB1 gene expression	Hassanpour et al. (2021)
DCM	Aerobic exercise	A motor treadmill running speed was increased until the speed reached 10 m/min	The heart and serum	HFD	The expression of P2X7R, NLRP3, caspase-1 and IL-1β	Chen et al. (2021)
DCM	Aerobic exercise	1 h of running on a motor-driven treadmill at 15 m/min at a 5° grade, 5 days/week for 15–16 weeks	Heart tissue and isolated coronary arteriole	A western atherogenic diet (0.2% cholesterol, 42% Kcal from fat)	NLRP3 inflammasome inactivation	Hong et al. (2018)
Hypertension	Aerobic exercise	High-intensity exercise	Serum and kidneys	High salt-diet	Upregulated β-OHB	Chakraborty et al. (2018)
Hypertension	Aerobic exercise	A treadmill for 14 weeks, 5 times a week and 60 min each time	Blood, vascular tissue samples, and mesenteric artery	Wistar Kyoto rats	NF-κB/NLRP3 inflammatory pathway	Luo et al. (2021)
Hypertension	Aerobic exercise	A 45-min running on a horizontal treadmill at 20 m/min, 5 days per week	Systolic blood pressure and the left ventricle tissues	Deoxycorticosterone-acetate and salt administration	NLRP3 inflammasome activation	Bal et al. (2022)
MI	Aerobic exercise and anaerobic exercise	10*1-min running intervals were separated by a 2-min rest	Blood samples from the heart and heart tissue	Isoproterenol	NLRP3 inflammasome	Ahmadi et al. (2022)
HF	Aerobic exercise	Low-, middle-, and high-intensity exercise	Serum and myocardial specimens	refer to Bedford’s motion load standard	NF-ĸB p65/NLRP3 inflammatory	Li et al. (2020)
HF	Aerobic exercise	2, 4, and 8 weeks of moderate-intensity aerobic exercise	Mouse heart tissues	Transverse aortic constriction	NLRP3/caspase-1/IL-1β signaling pathways	Ma et al. (2021)
HF	Aerobic exercise	A treadmill for 90 min/day and 6 days/week for 6 weeks at a velocity of 15 cm/s	Mouse hearts and primary neonatal mouse cardiomyocytes	Isoprenaline	NLRP3 inflammasome	Zhang et al. (2022)
HF	Aerobic exercise	A progressive, moderate intensity aerobic protocol	Human plasma*		IL-1β and ASC mRNA gene expression	Butts et al. (2018)
HF	Aerobic exercise	Three times a week, 60 min/session, for 8 months	Human serum*		Levels of TNF-α, IL-1β, IL-8, IF-γ and IL-10	Rodrigues et al. (2020)

*Indicated this study with cells or tissue from humans.

ASC, apoptosis-associated speck-like protein; AS, atherosclerosis; DCM, diabetic cardiomyopathy; FGF21, fibroblast growth factor 21; HFD, high-fat diet; HMGB-1, high mobility group box-1; Hcy, homocysteine; HHcy, hyperhomocysteinemia; HF, heart failure; IL, interleukin; β-OHB, β-Hydroxybutyrate; MI, myocardial infarction; NLRP3, NOD-like receptor protein 3; NF-κB, nuclear factor-κB; P2X7, purinergic receptor P2X, ligand-gated ion channel 7; AIN-93G, american institute of nutrition-93 growth).

The investigation of drugs targeting the NLRP3 inflammasome in clinical trials for CVD has been conducted. For example, the canakinumab anti-inflammatory thrombosis outcome study trial demonstrated the efficacy of IL-1-targeting therapy in preventing atherothrombotic events, indicating the potential of targeting the NLRP3 inflammasome (Ma et al., 2021; Ridker and Rane, 2021). However, the high cost of canakinumab limits its widespread use in the future. Additionally, long-term suppression of IL-1 signaling may have adverse effects such as increased susceptibility to infections and disturbances in immune homeostasis (Hettwer et al., 2022). Common cardiovascular drugs such as statins, beta-blockers, sodium-glucose cotransporter 2 inhibitors, and glucagon-like peptide 1 agonists may modulate NLRP3 inflammasome activity through different mechanisms, exerting protective effects in CVD (Terentes-Printzios et al., 2022). However, these classical medications carry certain side effects on gastrointestinal function and are prone to causing symptoms like hypotension and arrhythmias (Vaiciuleviciute et al., 2021). Interestingly, research suggests that exercise effectively modulates NLRP3 inflammasome activation, leading to improvements in AS and other CVD (Vaiciuleviciute et al., 2021; Terentes-Printzios et al., 2022). This therapeutic approach is relatively simple, feasible, and considered safe compared to using NLRP3 inhibitors and classical medications for CVD. Therefore, combining medication treatment with exercise intervention offers a potential strategy to improve the quality of life for patients.

## Figures and Tables

**Table:** 

**ASC**	apoptosis-associated speck-like protein
**AS**	atherosclerosis
**AT**	adipose tissue
**BMDM**	bone marrow-derived macrophages
**BP**	blood pressure
**CVD**	cardiovascular diseases
**CARD**	caspase recruitment domain
**DAMPs**	damage-associated molecular patterns
**DCM**	diabetic cardiomyopathy
**Drp1**	dynamin-related protein 1
**ET**	endurance training
**EP**	exercise preconditioning
**EE**	exhaustive exercise
**EEE**	excessive endurance exercise
**FGF21**	fibroblast growth factor 21
**FOXO1**	forkhead box transcription factor O1
**GSDMD**	gasdermin D
**NT**	n-terminal
**NOX4**	nicotinamide adenine dinucleotide phosphate oxidase 4
**HFD**	high-fat diet
**HMGB-1**	high mobility group box-1
**Hcy**	homocysteine
**HHcy**	hyperhomocysteinemia
**HIIT**	high-intensity interval training
**HF**	heart failure
**IL**	interleukin
**LRR**	leucine-rich repeat
**LICT**	low-intensity continuous training
**IR**	insulin resistance
**LPS**	lipopolysaccharide
**β-OHB**	β-Hydroxybutyrate
**MICT**	moderate-intensity continuous training
**MI**	myocardial infarction
**mtROS**	mitochondrial ROS
**HR**_**max**_	maximum heart rate
**VO**_**2max**_	maximum volume of oxygen
**NLRP3**	NOD-like receptor protein 3
**NLR**	nucleotide-binding domain-like receptor
**NF-κB**	nuclear factor-κB
**NEAT1**	nuclear paraspeckle assembly transcript 1
**PAMPs**	pathogen-associated molecular patterns
**PYD**	pyrin domain
**P2X7**	purinergic receptor P2X, ligand-gated ion channel 7
**ROS**	reactive oxygen species
**RT**	resistance training
**SHR**	spontaneously hypertensive rats
**TGN**	trans-golgi network
**TXNIP**	thioredoxin interacting protein
